# Mechanism of assembly of type 4 filaments: everything you always wanted to know (but were afraid to ask)

**DOI:** 10.1099/mic.0.001311

**Published:** 2023-03-22

**Authors:** Vladimir Pelicic

**Affiliations:** ^1^​ Laboratoire de Chimie Bactérienne, UMR 7283 CNRS/Aix-Marseille Université, Institut de Microbiologie de la Méditerranée, Marseille, France

**Keywords:** type 4 pili, type 2 secretion systems, pilin, type 4 filaments, pili, nanomachines

## Abstract

Type 4 filaments (T4F) are a superfamily of filamentous nanomachines – virtually ubiquitous in prokaryotes and functionally versatile – of which type 4 pili (T4P) are the defining member. T4F are polymers of type 4 pilins, assembled by conserved multi-protein machineries. They have long been an important topic for research because they are key virulence factors in numerous bacterial pathogens. Our poor understanding of the molecular mechanisms of T4F assembly is a serious hindrance to the design of anti-T4F therapeutics. This review attempts to shed light on the fundamental mechanistic principles at play in T4F assembly by focusing on similarities rather than differences between several (mostly bacterial) T4F. This holistic approach, complemented by the revolutionary ability of artificial intelligence to predict protein structures, led to an intriguing mechanistic model of T4F assembly.

## T4F: What’s in a name?

Type 4 filaments (T4F) are filamentous nanomachines centred on a polymer of type 4 pilins, assembled by a distinctive multi-protein machinery. The story of T4F starts with type 4 pili (T4P), one of the many types of unrelated pili identified so far. Pili – a term often used interchangeably with fimbriae – are defined as non-flagellar hair-like appendages, extending up to several micrometres from the surface of prokaryotic cells [1]. They mediate key functions such as adhesion, locomotion and gene transfer, thus physically connecting prokaryotes with their environment. Although they have been studied almost exclusively in diderm bacteria for decades, it is now clear that pili are also widespread in monoderm bacteria [2–4] and archaea [5]. Originally distinguished by their morphological features and/or the functions they mediate [6] – T4P owe their name to this early classification that listed seven pilus types – pili are now classified by their structures and the multi-protein machineries involved in their biogenesis [1, 7].

Two aspects of T4P are particularly fascinating. They are the only known pilus type present in diderm bacteria, monoderm bacteria and archaea [8, 9], and they have a bewildering functional versatility. T4P promote adhesion to biotic/abiotic surfaces, formation of microcolonies and biofilms, a form of crawling on surfaces (known as twitching motility because of the jerky and irregular motions of individual cells), protein secretion, mechanosensing (ability to sense and respond to mechanical cues) and DNA uptake. Incredibly, in some species, a subtype of T4P known as T4aP (the classification of T4P into subtypes will be introduced later in this review) is capable of mediating most, if not all, of these functions. A key step was the acquisition by T4aP of a potent motor for pilus retraction [10], which allows them to retract rapidly [11] and exert extremely high pulling forces [12, 13]. Overall, this versatility explains why T4P are critical virulence factors in numerous human pathogens, which makes them primary targets for the design of antivirulence therapies [14].

Much progress has been made in understanding the molecular mechanisms of T4P-mediated functions. This has been covered extensively elsewhere [15, 16] and will be touched upon only briefly in this review. T4P-mediated adhesion is most often mediated by pilus subunits with intrinsic adhesive properties [17–19], including modular pilins where adhesive domains not restricted to T4P biology have been ‘grafted’ onto a pilin [20, 21]. This ‘cut and paste’ strategy has apparently been used by many bacterial species to turn their T4P into sticky filaments [20]. In diderms, T4aP also display at their tip the non-pilin effector PilC/PilY1 (reflecting the different nomenclatures in different models) [22–25], which is a large modular protein mediating adhesion to a variety of receptors using different adhesion modules [26]. Another T4P function – perhaps the most widely studied [15, 16, 27, 28] – is twitching motility, mediated exclusively by T4aP that bacterial cells use as ‘grappling hooks’ to pull themselves forward [10]. The pilus attaches to a surface and the tension is sensed by the cell, which reels the pilus in by retraction, pulling itself towards the point of anchoring [29, 30]. T4P also promote the first step in natural transformation, DNA uptake, by acting as molecular ‘harpoons’ allowing the capture of DNA from the environment. T4P bind DNA via a pilin with intrinsic DNA-binding ability – the best characterized is ComP in *Neisseria meningitidis* [31, 32] – and then bring it close to the cytoplasmic membrane (CM) upon retraction [33].

In contrast, our understanding of the molecular mechanisms of T4P-mediated assembly remains poor, which I argue in this review could be improved by looking into the superfamily of filamentous nanomachines that T4P belong to. It has long been known that the defining features of T4P – polymers of type 4 pilins assembled by a distinctive multi-protein machinery – are shared by other filamentous nanomachines with different structural features and functions. Soon after their discovery in the late 1980s, it became obvious that type 2 secretion systems (T2SS) – widely used by diderm species to secrete fully folded protein effectors into the environment [34–36] – are closely related to T4P. T2SS use a similar multi-protein machinery to assemble short polymers of type 4 pilins, which play a key role in the secretion process [37, 38]. The competence (Com) pilus is another filamentous nanomachine discovered in the 1980s, which is related to T4P [39]. Com pili are used by monoderm bacteria to promote DNA uptake during natural transformation [40]. As shown originally in *Bacillus subtilis *, Com pili are polymers of type 4 pilins assembled by a multi-protein machinery sharing many components with T4P [41]. Another fascinating discovery came more recently from archaea, where the archaeal flagellum – sometimes referred to as ‘archaellum’ [42] – was found to be composed of type 4 pilins and assembled by a multi-protein machinery similar to T4P [43, 44]. This suggests that the archaeal flagellum is nothing else but a rotating T4P [45]. Later, it was found that archaea display a variety of filamentous nanomachines composed of type 4 pilins [46].

T4P and the above filamentous nanomachines share a common origin – they belong to the same superfamily – suggesting that fundamental mechanistic principles must be conserved for their assembly and functioning. This represents an enormous resource for people studying them, with endless opportunities for cross-pollination, where findings in one system fertilize others. Unfortunately, although this has happened on occasion (e.g. processing of pilins by a dedicated peptidase), it is too often marred by the natural human tendency to focus on differences.

Focusing on similarities is made even more difficult by a string of different nomenclatures. We use different names for the same genes in almost every system, e.g. *pilC* and *pilY1* encode the same protein (mentioned above) in *N. meningitidis* and *Pseudomonas aeruginosa *. Worse, we define the same proteins using different terms, hinting at differences that often simply do not exist. For example, the term ‘pseudopilin’ is used for T2SS pilin subunits, which derives from the definition of the short T2SS pilin polymers as ‘pseudopili’ (the prefix pseudo meaning filaments that look like T4P but are something else) [47]. It is true that T2SS filaments are different from T4P, i.e. they remain intra-periplasmic, hence the recently proposed name ‘endopilus’ would be more appropriate [48]. However, their constitutive pilin subunits are bona fide type 4 pilins and not mere lookalikes [49]. The term pseudopilin is a misnomer, since overexpression of the T2SS major pilin subunit is enough to promote assembly of surface-exposed filamentous appendages that are morphologically [50, 51] and structurally [52] indistinguishable from T4P. The pseudopilus issue also applies to the Com pilus because long surface-exposed filaments have never been visualized in the original model *B. subtilis* [41]. However, in *Streptococcus pneumoniae* Com filaments are bona fide pili, micrometre-long surface-exposed filamentous appendages that are morphologically indistinguishable from T4P [53, 54], and their major pilin has a 3D structure characteristic of type 4 pilins [55].

Several years ago, as a first step in attempting to federate/unify the field and underline the close phylogenetic relationship between the above filamentous nanomachines, we proposed to name the superfamily T4F [8]. A new name was needed because not all these nanomachines display surface-exposed filamentous appendages and could therefore not simply be called T4P. All the systems (including T4P) that share the following defining features – a filamentous polymer of type 4 pilins assembled by a distinctive multi-protein machinery – are T4F, regardless of their origin (bacterial or archaeal), biological function, or the length of the filamentous polymer.

## Natural history of T4F

As shown by their discovery in Archaea and in phylogenetically distant phyla of Bacteria, T4F are widespread. In fact, a first hint that T4F might be universal in prokaryotes came from the detection of the genes encoding their specific components in hundreds of bacterial and archaeal genomes [8]. This was confirmed in a remarkable study by Denise *et al*. [9]. The authors used cutting-edge phylogenetic methods to detect T4F-encoding genes in thousands of prokaryotic genomes, classifying them and analysing their natural history. It was shown that one, and sometimes several, T4F are present in every inspected phylum of Bacteria and Archaea. Besides confirming their close phylogenetic relationship, this analysis suggested that a T4F must have been present in the last universal common ancestor. This ancestral T4F then evolved and diversified upon separation of Bacteria and Archaea, through multiple gene acquisition, duplication, fission and deletion events.

Several phylogenetically distinct T4F clades were identified, each with specific gene profiles and organizations [9]. Six different T4F are present in bacteria: T4aP, T4bP, T2SS, Com pilus, MSHA (mannose-sensitive haemagglutinin) pilus, and Tad (tight adherence) pilus. All of these were previously described and studied to various extents. T4aP and T4bP are two subtypes of T4P defined long ago according to sequence features of major pilins (much longer leader peptides define T4bP pilins) and organization of genes involved in their biogenesis (scattered in T4aP versus clustered in T4bP) [56, 57]. These characteristics, although useful, were later shown not to be discriminatory, e.g. in *Streptococcus sanguinis* T4aP the major pilin subunits have very long leader peptides and are assembled by genes that cluster together [58]. Nevertheless, as confirmed by Denise *et al*. [9], T4aP and T4bP are distinct T4F clades, which differ by gene profiles and specific sequence signatures in some components. T4aP are found in almost all bacterial phyla – they are the most widespread T4F – whereas T4bP are only present in Proteobacteria [9]. T2SS and Com pili, which have been extensively characterized as already mentioned, were both found to be widespread but restricted to diderm and monoderm species, respectively [9]. In contrast, the MSHA pilus, found originally in *Vibrio cholerae* – a poorly characterized T4F that received its name because it haemagglutinates erythrocytes [59] – is only found in a few Proteobacteria [9]. Finally, the last system is the Tad pilus, which is also called Flp (fimbrial low-molecular-weight protein) pilus after its characteristic major subunit [60]. The Tad pilus – encoded by a genomic island acquired by horizontal gene transfer – has been well studied because it is essential for biofilm formation, colonization and pathogenesis in multiple bacterial species [61]. Although Tad pili have long been thought to be T4bP, they represent a distinct pilus subtype and were recently referred to as T4cP [62]. Denise *et al*. showed that T4cP are extremely widespread, as they are found both in archaea and bacteria [9].

The following evolutionary scenario has been proposed for bacterial T4F [9]. T4bP appear to be the most ancient system in bacteria. Since T4bP are only found in diderm species, the original T4F-containing bacterium possibly had two membranes. This is consistent with the notion that the last common ancestor of bacteria was a diderm from which monoderms arose by loss of the outer membrane (OM) [63]. It was thus proposed that the ancestral T4F machinery was composed of five proteins – pilin(s), prepilin peptidase (PPase), extension ATPase, platform and secretin – which will be described later. After the acquisition of the PilT retraction motor, a first split led to the emergence of Com pili and T4aP. Com pili arose upon loss of the OM, secretin and PilT, while T4aP arose by acquisition of the PilMNO proteins. Then, T4aP further diversified into T2SS (upon loss of PilT) and MSHA (upon loss of PilT and acquisition of MshN). Intriguingly, T4cP had a completely different evolutionary path. They originated in Archaea, apparently from the Epd pilus [43], and were acquired by Bacteria upon interkingdom transfer.

The unambiguous confirmation that T4F share a close phylogenetic relationship [9] offers the possibility to take two further steps to unify the field. We should (1) promote the use of the T4F term and (2) attribute a pilus subtype to the five bacterial T4F that assemble pili. Since the terms T4aP and T4bP have long been used, and T4cP should be used for Tad pili as recently suggested [62], it is tempting to name Com pili T4dP and MSHA pili T4eP. If other subtypes of T4P are discovered in the future, they should be attributed the next letters in the alphabet.

## T4F Paraphernalia needed for filament assembly

The close phylogenetic relationship between T4F suggests that there is a common mechanism for filament assembly, which must rely on a set of conserved components. In this section, as indicated by the title, the genes involved in T4F biogenesis in the different bacterial systems will be listed, briefly described, and compared, with the intention of revealing this conserved set of components.

### T4aP

T4aP are the best characterized T4F. The two major diderm models are *P. aeruginosa* and *N. meningitidis *, in which exhaustive genetic screens have identified all the genes involved in T4aP biology [64, 65]. The genes necessary for pilus biogenesis – denoted by the same *pil* mnemonic in these two species, but often with different letters – are scattered through the genome. The machineries are almost identical [66], with 15 conserved proteins (Table 1), the only difference being that piliation in *P. aeruginosa* requires the products of the *pilZ* [67] and *pilY2* [68] genes, which in *N. meningitidis* are not required or are absent [69], respectively. The T4aP machinery is conserved in all diderms. It is also very similar in monoderms, as shown in *S. sanguinis* [70], although it is simpler, as there are fewer minor pilins, and the components associated with the OM – absent in monoderms – are missing. It should be noted that there are often additional genes modulating T4aP-associated functions [71], although they are accessory for pilus biogenesis. Some are shared, such as the PilT retraction motor [10, 12], and others are species-specific, such as the minor pilins ComP, PilV and PilX in *N. meningitidis *, which are key for DNA uptake, adhesion and bacterial aggregation, respectively [17, 31, 72].

**Table 1.** Comparison of the proteins essential for filament biogenesis in bacterial T4F machineries*. The core proteins, implicated in filament assembly, are highlighted in light blue

	T4aP†	T2SS‡	T4bP§	T4cP¶	Com**
**Pilus components**					
Major pilin	PilE	PulG	TcpA	Flp1	ComGC
Minor pilins	PilH	PulH	TcpB	TadE	ComGD
	PilI	PulI		TadF	ComGE
	PilJ	PulJ			ComGF
	PilK	PulK			ComGG
Non-pilin tip-located effectors	PilC1/PilC2	PulA	TcpF		
**CM components**					
PPase	PilD	PulO	TcpJ	TadV	ComC
Extension ATPase	PilF	PulE	TcpT	TadA	ComGA
Platform protein	PilG	PulF	TcpE	TadB/TadC	ComGB
CM sub-complex	PilM	PulL	TcpR	TadG	
	PilN	PulM	TcpD	TadZ	
	PilO	PulC	TcpS		
	PilP				
**OM components**					
Secretin	PilQ	PulD	TcpC	RcpA	
Secretin-associated protein	PilF	PulS		RcpB/RcpC/TadD	

^∗^MSHA is not represented because it is too poorly characterized. However, the core proteins are conserved.

†*N. meningitidis* has been chosen as an example.

‡*Klebsiella oxytoca* has been chosen as an example.

§The TCP from *V. cholerae* has been chosen as an example.

¶The Tad pilus from *Aggregatibacter actinomycetemcomitans* has been chosen as an example.

^∗∗^ *S. sanguinis* has been chosen as an example.

The T4aP machinery – visualized by cryo-electron tomography (cryo-ET) in *Myxococcus xanthus* [73] – exhibits a multi-layered structure spanning the cell envelope from the cytoplasm to the OM, where the proteins necessary for T4aP biogenesis (Table 1) could be mapped. Six of these proteins are pilus components: five type 4 pilins (one major and four minor) and the PilC/PilY1 protein, which is not a pilin. The four PilHIJK minor pilins – also found in T2SS, where they interact to form a complex [74, 75] – are localized at the tip of T4aP, where they are capped by PilC/PilY1 [24]. In *S. sanguinis* T4aP, PilHIJK are replaced by PilABC, in which PilA is a structural homologue of the I subunit, while PilB and PilC are modular pilins displaying grafted domains with adhesive properties [20, 21].

The other proteins necessary for T4aP biogenesis (Table 1) are either at the CM or the OM. The PPase PilD, which processes prepilins to generate the pilin subunits, is an enzyme embedded in the CM. It functions on its own, as it can complete prepilin processing in the absence of other Pil components [76]. Six other components (PilF, PilG, PilM, PilN, PilO, PilP in the *N. meningitidis* nomenclature) were mapped to the base of the T4aP machinery at the CM [73]. Numerous studies have reported interactions between these proteins [77–85]. The last two components (PilQ, PilW) are involved in making a gated portal in the OM for the emergence of the T4aP on the cell surface [86]. PilQ belongs to the secretin family of giant pores, found in a variety of systems, allowing various substrates to traverse the OM in diderms [87, 88]. Structures of the T4aP secretin show a gated channel composed of 14 PilQ monomers, spanning the periplasm [89, 90]. PilW is an OM lipoprotein that interacts with PilQ [91] and is essential for the stability of the PilQ multimers [69, 92] and for T4aP functionality [93]. It was shown by cryo-ET in *Thermus thermophilus* that PilQ undergoes major conformational changes to make way for pilus extrusion [94]. The complex in the OM is connected to the CM complex via PilP, which interacts with both PilQ [95] and the PilMNO complex [96].

Three different approaches confirmed that not all of the above proteins are involved in filament assembly per se. Firstly, it was shown in several species that T4aP can be produced in the absence of some Pil proteins, provided that pilus retraction is abolished. For example, in mutants in *pilC*/*pilY1* and *pilQ* genes, piliation could be restored by a second mutation in *pilT* [86, 97–99]. Intriguingly, filaments assembled in the *pilQ pilT* mutant – in the absence of secretin – were trapped in the periplasm [86, 100]. This indicates that the ‘outside-in pathway’ proposed in *M. xanthus* – suggesting that the building of the T4aP machinery must initiate with the formation of the secretin portal before continuing inwards – is not generally applicable [101]. When used systematically in *N. meningitidis *, this genetic approach revealed that only 8 out of the 15 proteins required for piliation might be involved in filament assembly (PilD, PilE, PilF, PilG, PilM, PilN, PilO and PilP) [100, 102]. Secondly, using a synthetic biology approach, these eight proteins were shown to be sufficient for promoting pilus assembly in a nonpiliated heterologous host [102]. Thirdly, study of T4aP in the monoderm model *S. sanguinis* showed that the absence of OM components PilQ and PilW does not prevent T4aP assembly [70].

### T2SS

Although they are the only bacterial T4F that does not produce a surface-exposed pilus, T2SSs are the most closely related to T4aP [9]. The T2SS and T4aP machineries are almost identical (Table 1), except for a few differences. PulL corresponds to a fusion of PilM and PilN [103], which is consistent with the interaction between these proteins in T4aP [84, 85]. PulN is unique to T2SS, but is only found in a subset of species [38]. Although part of the complex at the CM [80, 104], PulN does not play a central role in secretion [105]. The last difference is the absence of PilC/PilY1 in T2SS, which in T4aP interacts with the PilHIJK complex at the tip [24]. This is perhaps not unexpected, as the HIJK complex, which is also present in T2SS [74, 75] – the two sets of proteins are even functionally interchangeable for piliation [106] – interacts directly with secreted effectors and is likely to play a role in their recognition [107]. Critically, the eight Pil proteins sufficient for pilus assembly in a heterologous host [102] are conserved in T2SS, which is consistent with a role in T4F assembly.

### T4bP

As shown by cryo-ET, the T4bP machinery [108] is similar to the T4aP machinery [73]. However, T4bP machineries are simpler, with 10–12 components [109, 110], which indicates that they are missing some T4aP components (Table 1). Firstly, the HIJK complex of minor pilins is absent, replaced by fewer minor pilins, which nevertheless appear to play the same role. For example, CofB in *V. cholerae* TCP (toxin co-regulated pilus) forms a tip-located trimer [111], which interacts with the secreted CofJ effector that is important for bacterial attachment [112]. Secondly, the MNOP proteins that form a complex at the CM are also replaced by fewer proteins. The comparative cryo-ET analysis of T4bP [108] and T4aP machineries [73], suggested that non-homologous proteins formed similar structures in the two systems. In particular, TcpRD appears to replace PilMNOP [73], which is consistent with the findings that TcpR interacts with the extension ATPase and is stabilized by TcpD [113]. Importantly, the eight T4aP proteins predicted to play a role in pilus assembly [102] are conserved in T4bP.

### T4cP

T4cP machineries are as complex as T4aP, with 14–15 components (Table 1) [114, 115]. The results of the comparative analysis confirm that T4aP proteins predicted to play a role in pilus assembly [102] are mainly conserved in T4cP. In brief, the conserved set of proteins includes major and minor pilins (TadEF replaces PilHIJK), a PPase (TadV) that lacks the methylation domain found in PilD, an extension ATPase (TadA) and, curiously, two platform proteins (TadB and TadC). Unlike other platforms that display a duplication of the platform signature motif (IPR018076 or IPR042094), TadB and TadC only have one. A phylogenetic analysis confirmed that TadB and TadC derived from an ancestor with two platform domains by an event of gene fission [9]. As in T4bP, the MNOP proteins are missing, but there are several specific Tad proteins that are predicted to localize to the CM, which could be part of the CM assembly complex, but this remains speculative.

### Com pilus

The Com pilus might hold the key to the unravelling of the mechanisms of T4F assembly. Indeed, with just eight components (Table 1) [116, 117] – five pilins (one major and four minor), PPase, extension ATPase and platform – it is the closest to the putative ancestral T4F machinery [9]. Strikingly, while the MNOP proteins are absent in the Com pilus, they are not replaced by other CM proteins as in T4bP and T4cP. Therefore, the complex at the CM involved in Com pilus assembly is only composed of two proteins: the platform and the extension ATPase. This suggests that the minimal T4F assembly complex consists solely of these two proteins, which invites the question, why are there additional proteins in the CM complexes in the above evolved T4F? A closer look at T4aP offer a possible explanation. It appears that there are multiple, mutually stabilizing interactions between the proteins of the CM complex [77–85]. Therefore, PilMNOP proteins might have been scored as essential for assembly [100, 102] only because they are necessary for stabilizing the other components of the assembly complex, i.e. the platform protein and extension ATPase.

## Focus on the four core proteins that are key players in T4F assembly

As discussed above, only four proteins – pilin(s), PPase, extension ATPase and platform protein – are found in every T4F system, no matter how simple or complex. It is thus likely that these four components – which are thus referred to as ‘core’ proteins – constitute the minimal machinery necessary to assemble T4F and were present in the last common ancestor. This is supported by the existence of a T4F consisting only of core components, i.e. the Com pilus involved in DNA uptake in monoderm species. In this section, the four core proteins that are key players in T4F assembly will be presented in detail.

### Type 4 pilins

**Fig. 1.**
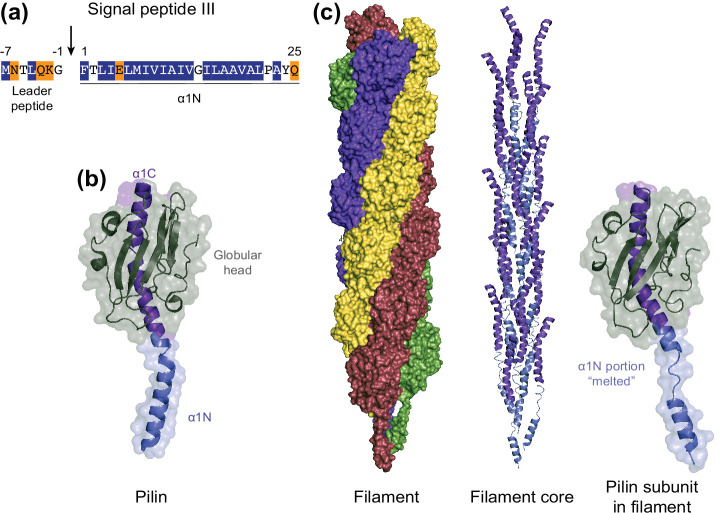
Type 4 pilins and T4F. *Neisseria gonorrhoeae* has been chosen as an example. (**a**)Prepilins are defined by an N-terminal SPIII motif [43]. The SPIII consists of two regions: a hydrophilic leader peptide, followed by a long stretch of hydrophobic residues invariably forming an α-helix (α1N) [49]. The leader peptide is cleaved by the prepilin peptidase (vertical arrow) [136, 137]. Hydrophilic residues are in orange, hydrophobic residues are in blue and neutral residues are unshaded. (**b**)Pilins share a characteristic ‘lollipop’ 3D architecture, first seen in the crystal structure of the *N. gonorrhoeae* major pilin (PDB 2IH2) [126]. A globular head (grey) – often built around an antiparallel β-sheet – is packed against the C-terminal half (α1C) of a long α-helix (purple) [49]. The protruding N-terminal half (α1N) of this α-helix (blue) corresponds to the hydrophobic portion of the SPIII. (**c**)T4F are helical assemblies of pilins. Left, surface view of the *N. gonorrhoeae* T4P structure (PDB 5 VXX) [129], where subunits P, P_+1_, P_+2_ and P_+3_ are coloured in burgundy, green, purple and yellow, respectively. Centre, ribbon view of the core of pilus structure composed of the α1-helices of the pilin subunits. The N-terminal (α1N) and C-terminal (α1C) halves of this α-helix have been coloured as in (b). Right, structure of a pilin subunit in the pilus. This shows that a portion of α1N is unfolded (melted) during filament assembly [129].

Type 4 pilins (Fig. 1) are the subunits of T4F [49]. T4F are essentially composed of one major pilin – although T4F with two major pilins have recently been described [70, 118] – and several minor pilins in much lower abundance. These proteins are synthesized as prepilins, which invariably display an N-terminal sequence peptide (SP) referred to as SPIII (to distinguish it from SPI in secreted proteins and SPII in lipoproteins) (Fig. 1a) [119]. Type 4 pilins can be identified by using dedicated programs such as PilFind [119] and SignalP [120], or by scanning a protein against signature databases such as InterPro [121]. In InterPro, the IPR012902 entry corresponds to the SPIII [49], which consists of two domains (Fig. 1a). The N-terminal domain (NTD) – known as the leader peptide – is rich in hydrophilic and neutral residues and invariably ends with one of the two smallest amino acids (aa): Gly or Ala. The leader peptide, which can be variable in length, is followed by a 20–25 residues-long stretch of mainly hydrophobic aa, with often, but not always, a Glu in fifth position [49]. When emerging from the ribosome, the SPIII is recognized by the signal recognition particle and targeted to the Sec machinery [122, 123], which ensures translocation of prepilins across the CM. Prepilins thus adopt their correct topology, with the leader peptide in the cytoplasm – because of what is known as the positive-inside rule [124] – and the long hydrophobic stretch in the membrane that acts like a trans-membrane (TM) domain [125]. This positions prepilins correctly for processing by the PPase (see below).

Despite often displaying little, if any, sequence homology besides their conserved SPIII terminus, pilins share a characteristic ‘lollipop’ 3D architecture [49], with a rounded globular head on the end of an α-helix ‘stick’ (Fig. 1b) [126]. The stick is an α-helix of approximately 50 residues (α1), whose protruding N-terminal half (α1N) corresponds to the hydrophobic stretch in the SPIII. The C-terminal half of α1 (α1C) is packed against the globular head, which is often built around an antiparallel β-sheet. However, there are variations such as in (1) major pilins from *Geobacter* species, where the stick and globular head are two different proteins [127]; (2) major pilins from Com pili in Firmicutes that display a purely helical globular head [55]; or (3) modular minor pilins with a variety of protein domains grafted onto a pilin via short linkers [20, 21].

As confirmed by multiple recent T4F structures [52, 118, 127–131], pilins are always staggered in a helical array, with their α1 forming the core of the filament and their globular heads exposed on the surface (Fig. 1c). Although the helical parameters – rise and azimuthal rotation between subunits – differ substantially between different T4F, these filaments share the same architecture [132]. They are held together primarily by extensive interactions within their hydrophobic α-helical core (Fig. 1c) [133]. Surprisingly, while, the α1 is a continuous helix in monomeric pilins, with a prominent kink at the end of α1N, there is loss of α-helical order in this portion of pilins within bacterial T4F (Fig. 1c) [52, 127–131]. The corresponding segment of α1N is thus somehow ‘melted’ (unfolded) upon polymerization. Consequently, the α1C runs roughly parallel to the filament axis, while the N-terminus of α1N is tilted toward the centre of the filament. In addition, this allows the formation of a salt bridge between negatively charged Glu_5_ in one subunit and the positively charged N-terminal amine of the adjacent subunit above, which is thought to neutralize these charges in the otherwise hydrophobic core of the filament and/or to drive subunit docking into a growing pilus [126, 128, 134, 135].

While it cannot be excluded that a T4F composed exclusively of one major pilin exists and might be discovered, the known T4F always contain minor pilin subunits in low abundance, which are either essential or dispensable for filament assembly. Usually, the minor pilins essential for filament assembly are more conserved – such as PilHIJK – and can be viewed as core components. These minor pilins interact to form a tip-located complex [20, 21, 74] capped by a subunit lacking the conserved Glu_5_. Since T4F are assembled from tip to base, filament assembly starts with this complex of minor pilins. In contrast, system-specific (non-conserved) minor pilins – such as ComP, PilV and PilX in *N. meningitidis* [17, 31, 72] – are dispensable for filament assembly and they are distributed along the length of the filaments [17, 72].

### Prepilin peptidase

**Fig. 2.**
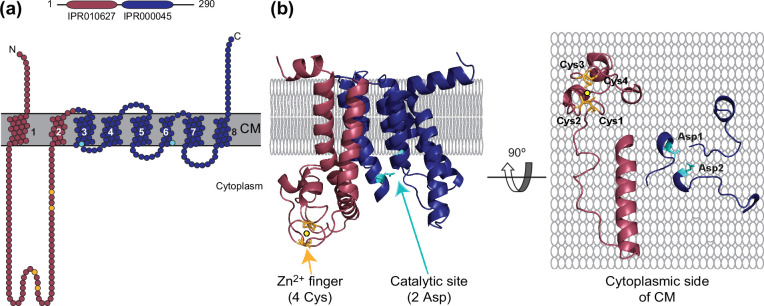
Prepilin peptidase. The *P. aeruginosa* PPase PilD has been chosen as an example [137]. (**a**)Bioinformatic analysis. Top, PPases are defined by an IPR010627 motif, which corresponds to the methylase domain (burgundy), and IPR000045, which corresponds to the peptidase domain (dark blue). Bottom, PPase topology consists mainly of eight TMs, some of which may not fully cross the CM. There is only one large cytoplasmic portion in the methylase domain (burgundy), which contains four conserved Cys residues (orange). The peptidase domain (dark blue) is mostly membrane-embedded and contains two catalytic Asp residues (light blue) [145]. The protein TM topology has been adapted from DeepTMHMM predictions [160]. The diagram has been generated using Protter [171]. (**b**)Highly accurate structure prediction using AlphaFold [151]. The pLDDT for *P. aeruginosa* PPase PilD is 92.2 %. Left, the structure in ribbon view has been placed in the CM according to its predicted TM domains. The methylase and peptidase domains are highlighted in colour, as well as the conserved Cys and Asp residues. The four Cys residues in the methylase domain form a zinc finger (the metal is represented as a yellow sphere), which probably explains PPase ability to bind Zn^2+^ [76]. Right, orthogonal view of the PPase showing the residues exposed at the cytoplasmic side of the CM, where the processing of prepilins is catalyzed.

Before they can be assembled in filaments, the SPIII in prepilins needs to be recognized and processed by a dedicated signal peptidase known as PPase (Fig. 2) [136, 137]. Unlike other signal peptidases, which have an active site on the extra-cytoplasmic side of the CM and cleave the entire SP, PPases are integral membrane proteins that only cleave the leader peptide in SPIII that is on the cytoplasmic side of the CM [125, 138], which means that their active site is on that side of the CM, very close to the membrane. This is compatible with the experimentally determined topology of PPases, which consist of eight TM regions and four small cytoplasmic loops (Fig. 2a) [139].

Often, but not always, processing involves subsequent methylation of the pilin new N-terminus [140]. PPases are thus readily identified by scanning databases such as InterPro, as bi-modular proteins with an NTD involved in N-methylation of pilins (IPR010627), and a C-terminal domain (CTD) involved in proteolysis of the leader peptide in prepilins (IPR000045) (Fig. 2a). There are, however, PPases with no methylase domain, such as in T4cP [141]. The functional significance of pilin methylation remains a mystery more than 40 years after its discovery [142], although it has been proposed to facilitate pilin membrane escape [143]. While the methylase domain contains four conserved Cys residues once erroneously thought to be important for proteolysis [144], the peptidase domain contains two universally conserved Asp residues that are critical for the cleavage of the leader peptide [145]. This was confirmed for multiple T4F in bacteria [58, 146, 147] and archaea [148], representing an example of ‘cross-pollination’. This suggests that PPases are aspartic proteases [145], which remains to be formally demonstrated.

How prepilins are processed by PPases remains poorly understood. However, no other protein is required since co-synthesis of the two proteins in a cell-free translation system leads to complete prepilin processing [76]. This study also showed that PPases bind Zn^2+^, which is required for S-adenosyl methionine-dependent methylation of pilins but is dispensable for cleavage of the leader peptide [76]. Critically, when mapped onto the PPase topology [139], the two universally conserved Asp residues that are key for cleavage are found at the interface between the CM and cytoplasm, while the four conserved Cys residues in the methylase domain are found in the first and largest cytoplasmic loop (Fig. 2a). This is compatible with these residues being part of two active sites sequentially catalyzing the processing of prepilins on the cytoplasmic side of the CM.

Unlike nearly all known aspartic proteases [149], PPases are not inhibited by pepstatin, a naturally occurring hexa-peptide [145]. Moreover, the only available PPase crystal structure for the archaeal FlaK [150], which consists solely of a peptidase domain, is apparently incompatible with the general acid–base mechanism for aspartic proteases. In this mechanism, two Asp residues coordinate a water molecule and activate it to perform a nucleophilic attack on the scissile bond of the substrate [149]. In FlaK, the two Asp residues do not face each other and are too distant to coordinate a water molecule [150]. However, using the ability of the artificial intelligence system AlphaFold to predict protein structures [151] – a recent revolution in structural biology [152] – highly accurate 3D structure predictions for PPases can be generated, consistent with all the above experimental data (Fig. 2b). The per-residue confidence metric called pLDDT is over 90 %. In brief, the predictions highlight a bundle of eight α-helices corresponding to TM domains, which allow positioning of the structure in the membrane. The methylase domain corresponds to the region encompassing the first two TM domains, whereas the rest of the protein corresponds to the peptidase domain (Fig. 2b). The conserved catalytic residues are on the cytoplasmic side of the CM, where prepilin processing occurs. Critically, the four Cys residues in the methylase domain appear to form a C4-type zinc finger (Fig. 2b), which explains the reported zinc-binding ability of PPases [76]. In addition, in contrast to FlaK [150], the conserved Asp residues face each other and are close enough to coordinate a water molecule (Fig. 2b). This strengthens the notion that PPases are classical aspartic proteases that use the general acid–base mechanism to cleave the leader peptide in prepilins [149]. The two Asp residues are likely to coordinate a water molecule, which would be activated by the first Asp to perform a nucleophilic attack on the prepilin scissile bond, generating a tetrahedral oxyanion intermediate stabilized by hydrogen bonding with the second Asp. Rearrangement of this intermediate would result in the cleavage of the prepilin leader peptide right after the last Gly or Ala residue.

### Extension ATPase

**Fig. 3.**
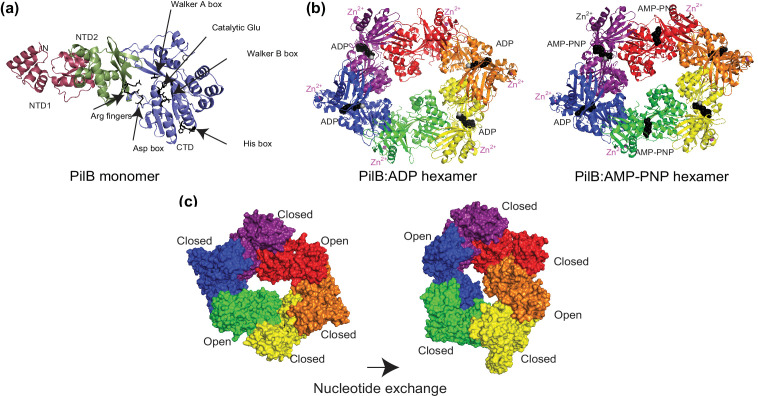
Extension ATPase. The ATPase PilB from *Geobacter metallireducens* has been chosen as an example [154]. (**a**)Structure prediction of a PilB monomer using AlphaFold [151], with a pLDDT of 88.99 %. This reveals N-terminal (NTD1 and NTD2) and C-terminal (CTD) domains. NTD1 is not found in all ATPases. Highly conserved residues/motifs [154], most of which are involved in binding and hydrolyzing ATP, are highlighted in black. These include Walker A and B motifs, a catalytic Glu, two Arg fingers, the Asp box and the His box. (**b**)Crystal structures of PilB hexamers bound to ADP (left) or AMP-PNP (a non-hydrolysable ATP analogue) (right), with each chain coloured (PDB entries 5TSG and 5TSH) [154]. NTD2 is facing up. NTD1 could not be built into the electron density [154]. ADP and AMP-PNP are shown as black spheres, while Mg^2+^ and Zn^2+^ are shown as grey and magenta spheres, respectively. (**c**)Clockwise rotation of PilB hexamers during nucleotide exchange. Two previously closed packing units open, while two open packing units close. This movement is transmitted to the platform protein.

Once pilins have been processed, they form a pool of subunits in the CM ready to be assembled into filaments. Polymerization occurs at the CM and proceeds from the tip to the base of the filament [73, 108]. For polymerization to occur, pilins need to be expelled/extracted from the CM and polymerized, which requires energy. Energy is provided by an extension (or traffic) ATPase in the cytoplasm, which forms hexameric ring-shaped structures with a central pore (Fig. 3). The extension (and retraction) ATPases belong to the family of AAA proteins (ATPases Associated with diverse cellular Activities) that undergo conformational changes upon ATP hydrolysis, which generates mechanical forces acting upon an interacting protein [153]. Several residues/motifs are highly conserved in extension/retraction ATPases, including Walker A and B motifs binding the β- and γ-phosphate moieties of ATP and Mg^2+^, respectively, a catalytic Glu for hydrolysis of the γ-phosphate, two arginine fingers, the Asp box and a His box [154].

Structurally, extension ATPase monomers consist of at least two globular domains on which the above conserved residues can be mapped (Fig. 3a), NTD and CTD, the latter of which binds and hydrolyzes ATP. The structural characterization of several extension ATPases revealed elongated hexamers (Fig. 3b) [103, 154–158]. The principal inter-chain contacts resulted from interactions between the NTD of one chain and the CTD of an adjacent chain, which together form one packing unit. Solving structures bound to different nucleotides/non-hydrolyzable analogues highlighted important differences in nucleotide binding and domain movements [154]. The interface between two packing units is the site of nucleotide binding [154]. Two successive ‘closed’ interfaces with bound nucleotides are followed by an ‘open’ interface, where no nucleotide is present (Fig. 3c). This pattern of one open for every two closed interfaces, which is required to maintain a closed ring, gives the hexamer its elongated appearance. Sequential ATP binding, catalysis and release were proposed to cause a clockwise rotation, about the axis parallel to the plane of the hexamer, and a thrusting up of the packing units that go from being closed to open. Some of the conserved residues move up by as much as 13 Å. A model was proposed to explain how these two motions could be transmitted to the platform protein to support the assembly of a right-handed helical filament [154].

### Platform protein

**Fig. 4.**
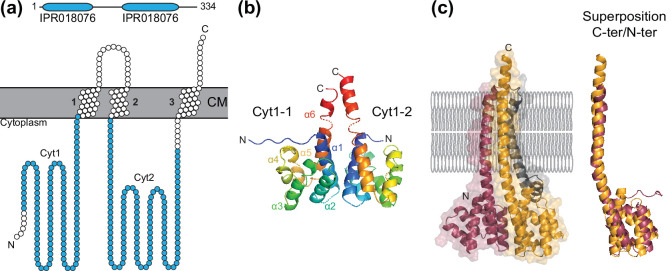
Platform protein. (**a**) Bioinformatic analysis. ComGB from *S. sanguinis* has been chosen as an example [55]. Top, platform proteins are defined by an IPR018076 motif, readily identified by searching InterPro [121], which is often present twice (blue rounded rectangles). The drawing is to scale. Bottom, topology of platform proteins with three TMs, defining two large cytoplasmic domains named Cyt1 and Cyt2. The IPR018076 motif in Cyt1 and Cyt2 has been highlighted in blue. The protein TM topology has been determined using DeepTMHMM [160]. The diagram has been generated using Protter [171]. (**b**)Crystal structure of EpsF from *V. cholerae* (PDB 2W7V) shows that Cyt1 is a six-helix bundle domain with a propensity for homodimerization [164]. Ribbon view of the EpsF homodimer in which each monomer (Cyt1-1 and Cyt1-2) has been coloured in rainbow spectrum from blue (N-terminus) to red (C-terminus). (**c**)Highly accurate structure prediction of the full-length platform protein using AlphaFold [151]. ComGB from *S. sanguinis* has been chosen as an example [55], and exhibits a 89.08 % pLDDT. Left, structure in the context of the CM. The structure in ribbon view has been placed according to its predicted TM domains [see (a)]. The two repeated domains are highlighted in colour. Right, superposition of the repeated domains, showing structural identity.

The platform protein – a poor name (because it is too generic) for the most poorly characterized core protein – interacts with the extension ATPase to transmit mechanical forces generated in the cytoplasm to processed pilins (Fig. 4). This integral membrane protein is thus the hub in the polymerization of pilin subunits into T4F, since processed pilins have no cytoplasmic portion and hence cannot interact directly with the extension ATPase. In fact, ‘T4F assembly hub’ could be a better name for this key, but poorly characterized, protein. Platform proteins are readily identified by the IPR018076 motif [121], which is most often present twice in the same protein (Fig. 4a). In T4cP, TadB and TadC have only one such motif [61], and were confirmed by a phylogenetic analysis to have derived from an ancestor with two domains by an event of gene fission [9]. The platform protein topology has been determined experimentally [159], revealing three TM regions connecting two large cytoplasmic domains (Cyt1 and Cyt2, which correspond to the duplicated portion of the protein) and a smaller periplasmic loop (Fig. 4a). This topology is consistent with the transmembrane topology predictions by dedicated programs, including the recent deep learning algorithm DeepTMHMM [160]. Many studies, using different experimental approaches and different systems, have shown that the platform interacts directly with the extension ATPase [78, 161, 162]. Interactions between the platform and pilins have also been reported [84, 163].

Structural information on the platform protein is limited to the Cyt1 domain, with several crystal structures for proteins from *V. cholerae* and *T. thermophilus* [164–166]. These structures revealed that Cyt1 folds as a six-helix bundle structure (Fig. 4b), with an extended final α-helix terminating at the point where the first and last TM helices begin. Each study further showed that the Cyt1 domain has a propensity to multimerize (Fig. 4b), forming dimers using slightly different interfaces [164–166]. This suggests that a multimeric assembly of the platform is likely to exhibit some flexibility. A very low-resolution 3D model of the *N. meningitidis* platform protein, using negative staining EM to visualize samples overexpressed and purified from *Escherichia coli* membranes, revealed an asymmetric bilobed structure approximately 125 Å in length and 80 Å in width [167]. The cytoplasmic portions of the protein correspond to the larger lobe, which is linked via narrower ‘waist’ region (the TM portion) to the smaller lobe corresponding to the periplasmic portion [167].

Although a full-length structure of the platform protein is yet to be determined, AlphaFold [151] is able to generate highly accurate predictions (Fig. 4c), which are consistent with the limited experimental data for this protein. These reveal a series of structural features that remained hidden for 40 years. The protein displays a ‘cherry pair’-like shape, where the two cherries correspond to the Cyt1 and Cyt2 domains (Fig. 4c). The stems correspond to three extended helices, which can be readily positioned in the CM, which exposes most of the protein in the cytoplasm. The central extended helix, which is broken in two by a prominent kink, connects two halves superposing almost perfectly (Fig. 4c). These two halves each encompass one extended helix, and therefore extend further than Cyt1 and Cyt2 domains. The platform has a tiny portion exposed on the extra-cytoplasmic side of the CM (Fig. 4c).

## Molecular mechanism of T4F assembly: a midsummer night’s dream

Can we combine the above findings into a coherent model for the molecular mechanism of T4F assembly, consistent with the current state of the art, which would go further than the current working model [154]? In that model, the sequential turnover of ATP in the packing units of the extension ATPase leads to a clockwise rotation of the hexamer and a perpendicular motion thrusting some packing units up [154]. This thrusting upwards motion is transmitted sequentially to the platform protein, which must interact with the motor via its duplicated Cyt domains, and then the pilin that is embedded in the CM. It was proposed that upon thrusting by the extension ATPase, the platform is rotated in 60° increments, thrusting pilin subunits upwards and then falling back in the membrane [154]. This motion supports the assembly of a right-handed helical T4F. This model was extended to the retraction motor PilT, where a counterclockwise rotation of the hexamer wrenches the platform downward towards the cytoplasm, rotating it in 60° increments to facilitate pilin depolymerization.

This extremely useful model is limited by the absence of structural information on (1) the platform, (2) the interface between platform and extension ATPase and (3) the interface between platform and pilins. The stoichiometry of the ATPase/platform complex, which remains unclear, is also an important limitation. Moreover, the model does not explain the melting of a segment of α1N in the pilins upon polymerization, which appears to be universal in bacterial T4F [52, 127–131]. Indeed, it is difficult to envision how pushing pilins out of the CM could lead to the observed loss of α-helical order in the spring-like α1N. Rather, the force would need to be exerted on the extra-cytoplasmic portion of the pilin, which needs to be ‘pinched’ by the platform and pulled to generate a force capable of unfolding α1N.

**Fig. 5.**
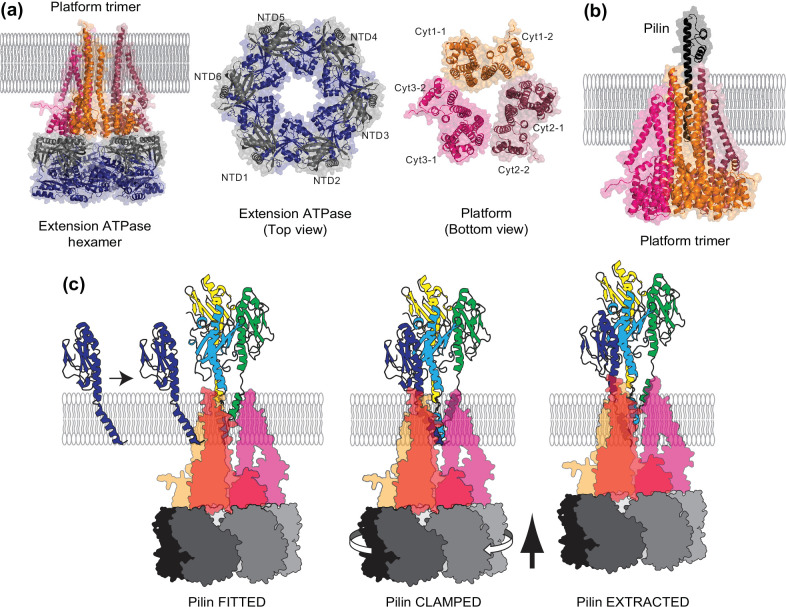
Atomic-resolution mechanistic model of T4F assembly. The components of the Com pilus from *S. sanguinis* have been chosen as an example because it is a minimal T4F [55]. The structures of the various complexes were predicted using AlphaFold-Multimer [168]. (**a**)Complex between the extension ATPase and the platform protein, in the context of the CM. Left, the structure in ribbon view has been placed in the CM according to its predicted TM domains (see Fig. 4a). Right, the extension ATPase is a hexamer. The NTD (grey) and CTD (blue) of each monomer have been highlighted. The platform protein is likely to be a trimer, which allows each of the six Cyt domains to interact with one ATPase subunit. (**b**)Complex between the platform protein and the pilin in the context of the CM. The platform trimer looks like a ‘drill chuck’, with the long α-helical stems of the three subunits forming a hollow shaft capable of holding the pilin. (**c**)Spindle motor mechanistic model for T4F assembly. The rotary extension ATPase motor powers a spindle formed by three copies of the platform protein. The platform is transparent to allow visualization of the pilins α1Ns. Left, a pilin subunit is FITTED in the spindle, which it enters laterally between two platform monomers. Centre, the clockwise rotation of the motor tightens the three-jaws drill chuck to CLAMP on the extra-cytoplasmic portion of the pilin α1-helix, right below the globular head. Right, the upward thrust of the extension ATPase is then transmitted to the pilin, which is EXTRACTED out forcibly from the membrane. This ‘clamping and extracting’ mechanism explains the unfolding of an α1N segment of pilins upon T4F polymerization in bacteria [52, 127–131]. The amplitude of the upward motion of the pilin matches that of the rise of the subunits in a one-start helix. Iteration of this process, by docking of the next pilin subunit at the base of the T4F, and ATP hydrolysis at the next active site in the hexameric ATPase, would ensure rapid T4F polymerization.

Using AlphaFold-Multimer, which is trained specifically for predicting 3D structures of protein complexes [168], I addressed the above limitations in the previous model, generating an intriguing mechanistic model of T4F assembly at atomic resolution (Fig. 5). It should be noted that this model remains purely speculative at this point – barely a ‘dream’, as indicated by the title of this section – as it is mainly based on AlphaFold predictions. First, the structure of the complex between extension ATPase and platform was predicted, which effectively solves a stoichiometry conundrum. The Com pilus was used because it consists solely of core components. Strikingly, an ATPase hexamer is likely to interact with a platform trimer, the NTD of successive ATPase subunits interacts with successive Cyt domains in platform subunits (Fig. 5a). This implies that the clockwise rotation of the hexamer, upon sequential ATP binding, catalysis and release, will be directly transmitted to the platform trimer to power a similar rotation. The thrusting up of the hexamer, described in structural studies [154], will similarly push on the platform monomers. How this motion is transmitted to the pilin is revealed by an AlphaFold structure prediction of the complex between one pilin and a platform trimer (Fig. 5b). The long α-helical stems of the three interacting platform subunits form a hollow shaft spanning the CM, in which the pilin fits readily. In this complex, the platform trimer looks strikingly like a ‘drill chuck’ with three jaws – each one corresponding to the long stem of one platform subunit – holding the pilin in its centre (Fig. 5b).

This drill chuck analogy leads to a mechanistic model for T4F assembly (Fig. 5c), consistent with everything we know about these filaments. The T4F assembly machinery is a spindle motor, in which a rotary hexameric extension ATPase motor [154] powers a platform protein spindle. This hollow shaft serves as a holder and a rotary drive for the pilin subunits. The process of assembly could be envisioned as follows (Fig. 5c). First, a pilin subunit is FITTED within the platform spindle by entering it laterally from the CM. The clockwise rotation of the motor is directly transmitted to the interacting platform, which acts a three-jaws drill chuck, tightening the CLAMP on the extra-cytoplasmic portion of the pilin α1-helix, right below the globular head. The upward thrust of the extension ATPase is then transmitted to the pilin via one or more of the three long α-helical stems in the platform subunits (Fig. 5c). Critically, unlike in previous models where pilins are somehow extruded/pushed out from the CM [154], in this new model the pilin is EXTRACTED/pulled out forcibly from the membrane. It will be important to show that this ‘clamping and extracting’ mechanism could generate forces sufficient to unfold an α-helix spring, which would explain the observed melting of the α1N segment of pilins upon polymerization in bacterial T4F (Fig. 5c) [52, 127–131]. The amplitude of the upward motion of the pilin would match that of the rise of the subunits in a one-start helix. Iteration of this process, by fitting of the next pilin subunit at the base of the T4F, and ATP hydrolysis at the next active site in the hexameric ATPase, would ensure rapid T4F polymerization. This could be further facilitated by the presence of three entrances for pilins into the hollow shaft – one between each platform monomer – allowing pilin subunits to be added sequentially to the growing filament at three sites around the circumference of the three-start helix.

## Concluding remarks

In this review, I tried to show that focusing on similarities between different T4F is a powerful approach for extracting meaningful information on poorly understood aspects of T4F biology, specifically filament assembly. Combined with a revolution in protein structure prediction, this enabled an intriguing mechanistic model of T4F assembly at atomic resolution to be proposed. This model is readily testable experimentally, which might lead to a dramatic improvement in our understanding of the mechanisms of T4F assembly. In turn, this would hold great promise for the design of innovative anti-T4F therapies.

Finally, the T4F field might be mature enough for a common effort to produce a homogeneous gene nomenclature, as done by the HUGO Gene Nomenclature Committee (HGNC), which assigns unique symbols to human genes [169]. Perhaps it is time to set up a T4F Gene Nomenclature Committee (T4FGNC), with nomenclature advisors? All the genes encoding the same proteins in different systems could be assigned a unique T4F symbol, without renaming them, e.g. T4FA (pilin), T4FB (extension ATPase), T4FC (platform) and T4FD (PPase)? The letters would in this case be from *P. aeruginosa *, where most of these genes have first been described [170]. Such an effort would improve scientific communication in the field tremendously and facilitate retrieval of information for newcomers and afficionados alike.
